# Radiotherapeutic management of brain tumours during the COVID-19 pandemic

**DOI:** 10.1017/S1460396920000394

**Published:** 2020-06-09

**Authors:** Rajesh Balakrishnan, Patricia Sebastian, Rajkrishna B, Jeyaanth Pulivadula Venkatasai, Selvamani Backianathan

**Affiliations:** Department of Radiation Oncology, Dr Ida B Scudder Cancer Centre, Christian Medical College, Vellore, Tamil Nadu, India

**Keywords:** brain tumors, COVID-19, guidelines, radiotherapy

## Abstract

**Aim:**

The coronavirus disease (COVID-19) pandemic is bound to put tremendous pressure on the existing healthcare system. This aim of this technical note is to help in triaging patients with brain tumours who are sent for radiotherapy during this pandemic and to provide safe and evidence-based care.

**Materials and Methods:**

Published data for this review were identified by systematically searching PubMed database from November 2007 onwards with the following Medical Subject Heading (Mesh) terms ‘Brain tumours’, ‘COVID-19’, ‘coronavirus’, ‘SARS-nCoV-2’, ‘Radiotherapy’, ‘Guidelines’ ‘hypofractionation’ using Boolean search algorithm. Articles in English language were reviewed.

**Results:**

We tried to apply the as low as reasonable achievable (ALARA) principle in triaging and management of patients for radiotherapy. We identified protocols which have hypofractionated regimens (reducing patient visits to hospital, time spent in treatment console) with similar outcomes when compared to conventional fractionated regimens and not overburdening the healthcare facility. We also identified the tumours for which we could safely avoid or delay the initiation of radiotherapy.

**Conclusion:**

Treatment decisions made during the COVID-19 pandemic rely on the safety first/do no harm principle and evidence-based prioritisation of cases for triage. This article is a tool to aid in triaging and prioritising brain tumour patient management. This is for consideration during the pandemic only and certainly not as a strategy for permanent practice change.

## Introduction

The World Health Organization (WHO) has declared the coronavirus disease (COVID-19) disease caused by the novel coronavirus as a pandemic on 11 March 2020 3 months after it was initially detected in Wuhan, China, in December 2019. In India, it was initially detected in Kerala on 30 January 2020 among the students who returned from China and subsequently the number of positive cases as on 29 April 2020 has risen to 30,000 with 940 deaths.^1^ At the same time, it had affected over 3 million people around the world with over 210,000 deaths.^2^

The current pandemic has led to many fundamental patient safety issues. Most importantly, the patients with cancer must leave their homes to visit the cancer clinic and thereby possibly expose themselves to infection. Another issue is these patients are susceptible to the infections as they are at an immunosuppressed state due to malignancy and anti-cancer therapy. Hence, patients with cancer are more predisposed to get infected with COVID-19. The question that we need to answer is should patients risk exposure to the Severe Acute Respiratory Syndrome novel Corona Virus 2 (SARS-nCoV-2) in order to receive treatment for cancer? The available evidence is limited, but suggests that the symptoms of COVID-19 are probably more severe in patients with cancer than for those without.^3^

It has also been shown that the mortality among the cancer patients is relatively high when compared to the normal population.^4^ The case fatality rate for cancer patients in that cohort was notably higher than non-cancer patients at 5.6% versus 2.1% in the whole sample.^4^ In another study by Liang et al., the patients with cancer were observed to have a higher risk of severe events (a composite endpoint defined as the percentage of patients being admitted to the intensive care unit requiring invasive ventilation, or death) compared with patients without cancer (39% vs. 8%), and the patients with cancer deteriorated more rapidly than those without cancer (median time to severe events, 13 days vs. 43 days).^5^

Tumours of the central nervous system (CNS) constitute about 1–3% of all malignancies, and they are a heterogeneous group as they differ widely in genetics and biology and they have various presentations and different clinical outcomes, depending on age, performance status, histology, surgery and postop adjuvant therapy received. Radiotherapy is an integral part in the management of brain tumour, either malignant or benign. Hence, it is vital to have guidelines on how to manage these patients.

We had decided to look at the impact of COVID-19 pandemic in neurooncology setting as it constitutes about 30% of our department clinics. Moreover, there are limited data in management of these tumours in the current scenario. Brain tumours usually require longer duration for surgery, require postop ICU care and these are the patients who are most likely to be neglected during the pandemic.

This article is to elucidate the radiotherapeutic management of patients with brain tumours during this COVID-19 pandemic. The various aspects about when to treat, when to safely avoid, how to triage and safety precautions to be taken during this period are detailed.

Our manuscript is intended to serve as a tool for consideration and certainly not as a strategy for permanent change in treatment patterns. The goal is to share options, as gathered collectively by our team, in both the management and surveillance of patients diagnosed with brain tumours during this time of global crisis in India.

In considering management of disease, we must recognise that in many centres across India and the globe, access to routine visits and surgery may be either completely restricted or significantly reduced. We must, therefore, formulate new options that we can offer our patients to address their disease limiting risk of exposure to the patients and healthcare workers.

## Universal Precautions

Current evidence suggests that COVID-19 is spread by droplets and has an incubation period of 1–14 days. In a situation, such as now, when WHO has declared COVID-19 as a pandemic, even though India has not entered into community spread phase, it is necessary to consider each and every patient whom we treat to be as potentially positive until and unless it has been proven. Hence, it is imperative that we take necessary steps and precautions while providing care to all our patients.

The guiding principle of radiation safety is ALARA which stands for ‘as low as reasonably achievable’. This principle means that even if it is a small dose, though it may not have much chances of obvious effect, one should try to avoid it.^6^ The same ALARA principle needs to be applied for patient care too during this scenario. Screening by front desk staff upon check-in and triage with nursing and physicians is necessary in order to protect the patients and to prevent possible exposures in both waiting rooms and treatment vaults from infected individuals.

## Time

We need to reduce the number of patient visits to the hospital and time spent in the treatment facility. This could be achieved in the ways mentioned below.

1. Hypofractionated schedule whenever safely possible.
2. Avoid radiation (RT) whenever possible.
3. Do image verification on a necessity/protocol basis and not every day.
4. Maintain time and schedule patients for treatment to avoid crowding at waiting room.

## Distance

1. Follow social distancing norms as advised by the government while making patients wait for their radiotherapy schedule.
2. Limit the number of staff/healthcare personnel in the treatment console area.

## Shielding

1. Wear appropriate personal protective equipments (PPEs) as per the national standards; the patients should wear mask during waiting time.
2. Provide disinfectant/sanitisers/soap and water with hand wash facility for the patients at the entrance of treatment facility.
3. Clean/disinfect the treatment couch and common immobilisation devices at regular intervals (after every patient) and immediately when it appears soiled. This is important as the treatment machine is shared by many patients.
4. Do not place immobilisation masks one over another and make arrangements to keep them separately in separate pigeon holes whenever possible.

### Specific scenarios

The recently published rapid COVID-19 NICE (National Institute for Health and Care Excellence) guidelines say that the patients should be treated with radiotherapy only if it is ‘unavoidable’, and even then the ‘shortest safe form of treatment’ should be used. The guidance also advised that radiotherapy should be avoided if the evidence suggested that there would be ‘little to no benefit or if an alternative treatment is available’ or should be deferred if clinically appropriate.^7^

**Figure 1.**
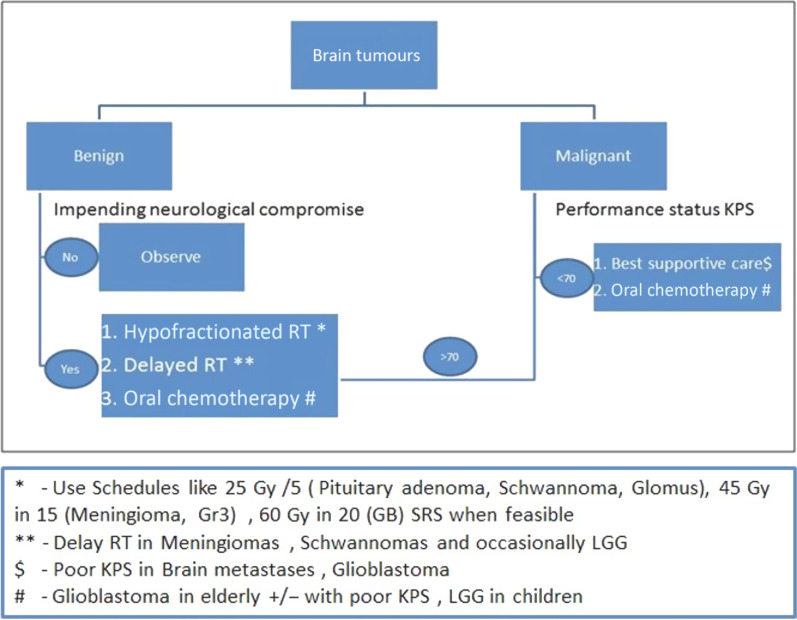
Treatment decision-making algorithm for patients with brain tumours during COVID-19 pandemic.

We also recommend discussing the patient’s overall prognosis, survival estimates and goals of care with the patient and his/her family and neurosurgeon prior to determining a radiation plan with validated prognostic models available in the literature. For patients with an estimated life expectancy of days to weeks, best supportive care with medical therapies alone is encouraged. The treatment decision-making algorithm proposed during the COVID-19 pandemic is given in Figure 1. The suggested treatment options for brain tumours^8–23^ are enlisted in Table 1.

**Table 1.** Suggested treatment options for brain tumors during the COVID-19 pandemic

Indication	Treatment strategy during COVID-19 pandemic	References
Brain metastases	For solitary/limited brain metastases with good DS-GPA—offer single-fraction frameless SRS Hypofractionated RT—30–35 Gy in 5–6 fractions For multiple brain metastases—whole-Brain RT—hypofractionated regimen (20 Gy in 5 fractions)	8–10
Spinal cord compression	Hypofractionated regimens 8 Gy in single fraction/20 Gy in 5 fractions	11
Arterio venous malformations	Avoid SRS or hypofractionated SRS Can safely postpone treatment	12,13
Meningiomas	Grade 1, Grade 2 meningiomas ○ Can delay start of RT ○ Hypofractionated RT (25 Gy in 5 fractions) Grade 3 ○ Delay start of RT or ○ Hypofractionated RT (45 Gy in 15 fractions)	14
Schwannomas	Delay start of RT If any neurological compromise could try frameless SRS/hypofractionated RT—25 Gy in 5 fractions	15
Low-grade gliomas	Delay RT and offer RT at progression Children—can try less intensive chemotherapy regimens in consultation with paediatric oncologist Chemotherapy with Temozolamide is a safe option in view of limited side effects	16,17
Glioblastomas	Elderly with poor KPS/unmethylated—best supportive care and Omit RT Elderly with poor KPS/methylated—TMZ only/RT—34 Gy in 10 fractions or 5 Gy weekly × 6 weeks Younger patients good KPS—hypofractionated RT—60 Gy in 20 fractions (SIB technique) with concurrent temozolamide. Try to start RT with 4–6 weeks after surgery	18–20
Medulloblastoma	Do not delay initiation of treatment beyond 4–6 weeks after surgery. Could start with posterior fossa boost and then could switch over to craniospinal RT with VMAT/IMRT which has lesser chances of haematological toxicity. Can omit concurrent chemotherapy if more complications anticipated.	21,22
Cystic craniopharyngiomas	Do not delay treatment. For all post-operative patients, start on RT as early as possible as the cysts could reaccumulate and cause visual compromise	23

### Care of patients during treatment and follow-up

For patients requiring care while receiving RT, visits could be arranged with the designated rotating radiation oncology healthcare providers during the day. Whenever face to face outpatient consultations are done, we need to ensure that all patients, caregivers and healthcare professionals adhere to institutional policies on social distancing, hand washing, using appropriate PPE to reduce the risk of exposure of staff and patients.

We also recommend teleconsultations and use of photographs sent via emails to assess the skin reactions and if needed could send drug prescriptions to patients.^24^ The patients who are on routine follow-ups need to be advised to postpone their follow-up visit by another 3–4 months. Our experience on such methods showed that the patients were extremely happy and were relieved of the stress that they do not need to come to the hospital unless it is necessary.

## Corticosteroids Usage

Steroids are most commonly used among brain tumour patients receiving radiotherapy. There have been reports stating that non-steroidal anti-inflammatory drugs (NSAIDs) and corticosteroids may exacerbate symptoms in COVID-19 patients. But some evidence has also showed that corticosteroids may be beneficial if utilised in the early acute phase of infection. Given the current availability of literature, we should exercise caution while using corticosteroids until further evidence emerges.^25,26^

## Conclusion

Throughout the COVID-19 pandemic, it is important to support our patient’s emotional wellbeing and ensuring that adequate psychosocial support systems are in place would be more important than ever. We should also not forget that safety is of foremost importance and all measures to prevent spread/transmission of COVID-19 disease such as provision of adequate and appropriate PPEs to the healthcare workers and ensuring availability of hand sanitisers. Treatment decisions made during the COVID-19 pandemic rely on the safety first/do no harm principle and evidence-based prioritisation of cases for triage. The 2019-nCoV outbreak in India is ongoing at the time of writing, with lockdown procedures and border controls being employed by the Indian government, as well as neighbouring countries. We are continuously reviewing our measures in response to the dynamic situation, in order to provide efficient and safe care for our patients.
